# Soft Drink Consumption in Young Mexican Adults Is Associated with Higher Total Body Fat Percentage in Men but Not in Women

**DOI:** 10.3390/foods9121760

**Published:** 2020-11-28

**Authors:** Cesar Campos-Ramírez, Víctor Ramírez-Amaya, Liliana Olalde-Mendoza, Jorge Palacios-Delgado, Miriam Aracely Anaya-Loyola

**Affiliations:** 1 Program of Biological Science, Department of Natural Sciences, Autonomous University of Queretaro, Av. de las Ciencias S/N, Juriquilla, Queretaro 76230, Mexico; cezzar-5@hotmail.com (C.C.-R.); lolalde13@alumnos.uaq.mx (L.O.-M.); 2 Instituto de Investigación Médica Mercedes y Martín Ferreyra INIMEC-CONICET-UNC, Friuli 2434, Colinas de Vélez Sarsfield, Córdoba 5016, Argentina; vramirezamaya@immf.uncor.edu; 3 University of Mexican Valley-Campus Juriquilla, Blvd. Juriquilla 1000 Querétaro, Querétaro 76230, Mexico; jorge.palaciosd@uvmnet.edu; 4 Department of Natural Sciences, Autonomous University of Queretaro, Av. de las Ciencias S/N, Juriquilla, Queretaro 76230, Mexico

**Keywords:** soft drinks, intake, body fat, metabolic variables, young Mexicans

## Abstract

A high consumption of soft drinks (SDs) has been linked with the development of anthropometric and metabolic alterations. We evaluate the association between SD consumption and some anthropometric and metabolic variables. This study is an observational study, using a sample of 394 university students, of which 158 were men (40.1%) and 238 women (59.9%), between 18 and 30 years. An SD intake questionnaire provided the consumption of different SDs. The participants’ weight, height, and waist and hip circumferences were collected. Metabolic biomarkers were analyzed. The average intake of caloric SDs (CSDs) was 1193.6 ± 1534.8 mL/week and 84.5 ± 115.02 mL/week for non-caloric SDs (NCSDs). Sex differences were found in the amount of SD consumption and these statistical differences were driven by those men subjects with a high total body fat percentage (TBF%). In men, correlations were found between the intake of CSDs and the body mass index, waist and hip circumferences, TBF%, and visceral fat percentage. In woman, a correlation was found with glucose and triglycerides. The prediction model revealed that the intake of CSDs predicts TBF% and low-density lipoprotein only in men. A high amount of CSD consumption in men was associated with a high TBF%, and this may be predictive of future development of metabolic abnormalities.

## 1. Introduction

Obesity is characterized by an excessive amount of adipose tissue, which is the main risk factor for the development of metabolic complications [1,2]. The prevalence of obesity has increased significantly worldwide: between 1980 and 2013, overweight and obesity increased from 28.8% to 36.9% in adult men, and from 29.8% to 38.0% in adult women [3]. The same trend is observed in children and adolescents [3]. In 2016, the Mexican National Health and Nutrition Survey (ENSANUT) reported a combined prevalence of overweight and obesity of 69.9% in males and 72.2% in females [4].

One of the main factors for the development of overweight and obesity are a hypercaloric diet, particularly those were refined carbohydrates predominate [5,6]. It has been observed that a high consumption of sugar-sweetened beverages (SSBs) are one of the major contributors of energy imbalance in young people [7,8,9]. In Mexico, soft drinks (SDs) are consumed daily by 74% of the population with an average of 115 L per person each year [8]. SDs are sweetened mainly with high-fructose corn syrup (HFCS), which contributes to a high amount of caloric intake [10]. It is important to note that HFCS is widely used in the production of beverages since it has a higher sweetening capacity than sucrose and its cost is more profitable [11].

In humans, the intake of fructose-sweetened beverages, together with a meal, induces a significantly lower secretion of the anorexigenic signals insulin and leptin, while the secretion of the orexigenic hormone ghrelin is more intense and lasts for a longer time as compared to the intake of glucose-sweetened beverages with the same meal. This metabolic response can further stimulate food intake [12] and possibly promote weight gain, too [13]. In agreement with these results, overweight and obese adult subjects, obtaining 25% of their daily energy requirements by either glucose- or fructose-sweetened beverages, over 10 weeks showed significantly higher visceral fat, increased hepatic de novo lipogenesis, higher 23-h postprandial triglycerides, and a significant decreases in insulin sensitivity [14]. More recently, a significantly linear dose-response between lipid/lipoprotein risk factors and the consumed amount of SDs containing HFCS has been observed [15]. In young adolescents consuming SSBs, a higher Body Mass Index (BMI) was found in those consuming both sugar- and HFCS-sweetened beverages, as compared with people who consume sugar-free or natural juice beverages [16].

The consumption of beverages sweetened with sugar and/or HFCS confers a high glycemic load [17,18] and also poor satiating properties [19], stimulating food intake. The estimated consumption of SSBs (including SDs products and processed fruit juice), measured by a food-frequency questionnaires (FFQ), showed that the higher the servings per week intake of these products, the higher the homeostatic model assessment and insulin resistance (HOMA-IR), also increasing the incidence of prediabetes in 46% of the subjects across a 14-year follow-up [20]. Moreover, in a worldwide study, a multivariate linear regression found a significant association between SSB consumption with overweight, obesity, and diabetes prevalence [21]. This association was also observed for other metabolic disorders, such as osteoporosis and certain types of cancer [8,21,22,23]

Due to the adverse effects of SSB consumption on health, nowadays more and more individuals are choosing alternatives, such as those beverages containing low or no calories. These alternative beverages are usually sweetened with artificial non-nutritive sweeteners (NNSs), such as saccharine, acesulfame, aspartame, and sucralose, among others. NNSs, in addition to reducing or excluding calories, have the feature of preserving or even enhancing the sweetness palatability [24].

Several studies have found that consumption of non-nutritive-sweetened beverages (NNSBs) is associated with the development of metabolic alterations [25,26] due to changes in the gut microbiome and associated to NNSs [27], but also to high sucrose [28] and HFCS consumption [29,30]. Artificial and natural NNSs are bacteriostatic and inhibits anaerobic bacteria in the gut [31,32]. Alterations in the gut microbiome tend to increase gut permeability and metabolic and immunological abnormalities [33], finally leading to the development of overweight and obesity in the consumer population [34]. Summarizing, the consumption of SDs, including both SSBs and NNSBs, contribute in different ways to the alteration of metabolism and the development of overweight and obesity.

Mexico is the largest consumer per capita of SDs in the world [35], but there are no studies linking this SSB consumption with anthropometric and metabolic variables in the young population. In this study, we evaluate the association of the intake of SDs and some anthropometric and metabolic variables in a young Mexican population. Our aim was to determine in a Mexican freshmen population the possible association of SD consumption with anthropometric and metabolic variables. It was found that caloric soft drink intake in the young Mexican population is higher in men than in women and is associated with a higher total body fat percentage in men while in woman it correlates with lipid metabolism signals.

## 2. Materials and Methods

### 2.1. Study Design

This is an observational, cross-sectional, and single time-point study, where the subjects were recruited by a non-probabilistic sample from the Autonomous University of Queretaro (UAQ, Santiago de Querétaro, Mexico) located in central Mexico during September to November 2018.

### 2.2. Subjects

A total of 700 freshmen were invited to participate in this study. The invited subjects were students attending the UAQ and participating in the university health program (SUSALUD for its acronym in Spanish), which is a comprehensive health program that performs an integral nutritional and health assessment of freshmen students. The inclusion criteria were (a) to be a freshmen-enrolled student of the UAQ and participating in the SUSALUD program; (b) to have signed the informed consent letter; (c) to attend the instructional talk for the study; and (d) to attend to the clinic for collecting the anthropometric data, a fasting blood draw, and an SD intake questionnaire (SDsIQ). The exclusion criteria were (a) to present any already diagnosed disease, a clinical condition (such as prostheses or electrical devices such as pacemakers); (b) to skip either the anthropometric or blood sampling; and (c) a null or partial SDsIQ. Participants enrolled voluntarily and did not obtain any type of remuneration, either monetary or in any other way, for their participation in the study. A written and signed informed consent letter was obtained from each subject. The study was approved by the ethics committee of the Department of Natural Sciences UAQ, with registration number 98FCN2017.

The subjects who met the eligibility criteria described above and agreed to participate in the study by signing the informed consent letter were cited at the Nutrition Clinic of the Department of Natural Science for an informative session a day prior to their nutritional evaluation. Data collected at the appointment included anthropometric, body composition, and blood sampling for the biochemical analysis, as well as the SDsIQ; 394 participants completed all the evaluations and were included in the statistical analyses.

### 2.3. Data Collection

#### 2.3.1. Instructional Session

For their first appointment, all participants were instructed to adhere to fasting conditions for the evaluation at the Nutrition Clinic. They were also advised to wear light clothes and to remove all metallic accessories for the body composition assessment. They also had to bring the answered SDsIQ.

#### 2.3.2. Anthropometric Data

All measures were performed by a trained nutritionist. Weight, height, waist, and hip circumferences were taken in duplicate following the standard procedures of the World Health Organization [36]. Height measurement was performed with a stadimeter (Holtain Limited, Crosswell, Crymych, Pembs). Waist circumference was measured by placing a measuring tape (Lufkin W606PM) on a line that is midway between the upper iliac crest and the lower costal edge, at the end of a normal expiration, with these measurements the indicators waist-to-hip ratio (WHR) and waist-to-height ratio (WHTR) were obtained. Weight determination and body composition data were measured using a multifrequency bioelectric impedance device (Seca mBCA 515, model 0123; Hamburg Germany). We used for this study only the total body fat percentage (TBF%) and visceral fat percentage (VF%). Diagnosis criterion for TBF% was done according to McCarthy et al. [37] for participants 17–18 years old, and according to Gallagher [38] for participants ≥19 years old; the classification of TBF% were normal, moderately high, and high.2.3.3. Biochemical Analysis

A 12-h fasting blood sample was drawn by a phlebotomist in 5.0 mL Vacutainer SST II plastic tubes with gel-separation gel to obtain serum for the analysis of glucose, total cholesterol (TC), triglycerides (TG), high-density lipoprotein (HDL), low-density lipoprotein (LDL), and very-low-density lipoprotein (VLDL). The biochemical determinations were carried out in duplicate in automated Mindray BS 120 (Medical International Limited, China) equipment, using in each run the Spintrol human calibrator serum (SPINREACT S.A./S.A.U., REF: 1002011 Girona, Spain) and standard serum controls (high REF: 1002210 and normal values REF: 1002210) to assure the precision and quality of the determinations. For the biochemical determinations, two fasting blood samples were taken from each participant.

#### 2.3.3. SDsIQ

The SDsIQ asked the participants whether or not they consume any of the different SDs available in the Mexican market. The SDsIQ include both the caloric and non-caloric version of every SD. It also allows the participant to select the flavor of the SDs (cola, apple, orange, grape, grapefruit, etc.). The SDsIQ gives the subjects the choice to select the serving size (30 mL, 250 mL, 355 mL, 600 mL, 1 L, or 1.5 L), based on the commonly available drinking containers or commercial presentations of the different products. Finally, the SDsIQ also allows the participant to select the frequency of consumption considering the past month (daily, weekly, monthly, or never).

This SDsIQ was based upon several other food frequency questionnaires that have been properly validated previously [25,39,40,41,42]. The SDsIQ was develop by a panel of experts in nutrition and food and beverages consumption from the UAQ in conjunction with the co-authors of this study. It was previously used in a master’s degree thesis. The list of products was selected according to the availability of such products in the local market. The frequency choices intend to provide flexibility for the subject, in order to account for the different SD products and consumption frequency habits. To quantify the amount of SDs consumed, the responses from the SDsIQ were transformed into mL/week using the frequency and serving size for each type of SD. The preferred type of SD was also evaluated by the mean of the higher amount of intake. Finally, three indicators were built: (1) CSDs (sum of the individual intake amounts of each of the SDs with caloric content); (2) NCSDs (sum of the individual intake amounts of each of the SDs without caloric content); and (3) total SD intake (sum of the previous two). 

### 2.4. Statistical Methods

Statistical analysis of the data was done using the SPSS 22 program. Descriptive statistics for the anthropometric and metabolic variables, as well as for the SD consumption data were performed. Data are presented in tables as the mean ± standard deviation or as percentages. Comparisons were made between the means of all the variables analyzed according to sex, and one-way analysis of variance (ANOVA) was performed to assess whether there is a difference between both sexes. A Spearman Rho correlation analysis was used to assess the association between CSD intake and the anthropometric and metabolic variables.

The relation between the TBF% diagnostic and CSD consumption was assessed by ANOVA in men and women. Analysis of quartiles was done to classify participants according to their CSD consumption. Differences between TBF% according to quartile class and sex was performed by one-way ANOVA. To estimate the relationship between CSD intake and the metabolic variables, a bivariate linear regression model was used. CSD consumption was compared between sexes, separated by the TBF%. We also compared TBF% by separating the subjects into “quartiles” of CSD consumption and comparing between men and woman by a one-way ANOVA. 

## 3. Results

### 3.1. Subjects

The 394 participants were from the faculties of Chemistry (22%), Engineering (15.5%), Languages and Letters (16.3%), Natural Sciences (26.4%), and Political Sciences (19.8%), from which 40.1% were men (*n* = 158) and 59.9% women (*n* = 238). The average age of the participants was 19.05 ± 1.97 years.

### 3.2. SD Intake

The average total SD intake was 1277.9 ± 1655.1 mL/week. Participants were found to consume mainly CSDs with an average consumption of 1193.2 ± 1534.9 mL/week, while NCSDs had a much lower intake with a mean of 84.6 ± 115.0 mL/week. The more frequently selected portion was 250 mL (1 glass) with a 43.5% preference, followed by 600 mL (24%) and 355 mL (24%). It was found that 49.6% of the participants preferred the cola flavor, followed by apple, lime-lemon, and orange flavor, with 12.9%, 8.4%, and 4.3% respectively. The remaining 24.8% was distributed in the other 12 different flavors. Significant differences were found between men and woman according to CSD intake; women reported to consume less CSDs in comparison to men (Table 1). The same trend was observed based on the flavor, the cola flavor being the most preferred by the participants, independent of sex. There were no significant differences between men and woman intake regarding to NCSD intake, although woman drink twice the amount of these products compared to men. Finally, when evaluating total SD intake, a significantly higher consumption was found in men (about 1.5 times the amount consumed by women). No differences were found in the CSD and NCSD intake based on the BMI for either men or women (data not shown).

### 3.3. Anthropometric and Metabolic Characteristics

As expected, there were significant differences in anthropometric variables: men presented a significantly higher weight, height, and waist circumference compared to women. However, the BMI mean was not statistically significant according to the sex of the participants. The higher waist circumference in men, indicating fat accumulation in the abdominal region, was confirmed with a significantly higher value of visceral fat found in men obtained from the body composition analysis. Additionally, TBF% was higher in women compared to men, which is also a common finding linked to the sex of the participants. According to the BMI classification, men presented a 3.7% prevalence of being underweight, 63% were normal weight, 24.4% were overweight, and 8.9% were obese. For women there were no underweight subjects; the prevalence for normal, overweight, and obesity were 66.2%, 24.8%, and 9%, respectively.

Consistent with the anthropometric data, men presented higher mean values for glucose, TG, and VLDL; all these biomarkers are associated with risk of cardiovascular disease. In contrast, women presented significantly higher values for HDL compared to men, which is a clear difference expected depending of sex (Table 2).

### 3.4. Correlation between CSD Consumption and the Anthropometric and Metabolic Variables

Statistically significant positive correlations (Table 3) were found between the amount/week of CSD consumption and the anthropometric variables BMI, waist, hip, WHR, WHTR, TBF%, and VF%, as well as with the metabolic variable glucose in men (*p* < 0.01), while in woman mild but significant positive correlations were found between the amount/week consumption of CSDs with the metabolic variables TG and VLDL (*p* < 0.05).

### 3.5. Comparison of CSD Consumption Based on TBF% Diagnosis

**Figure 1:**
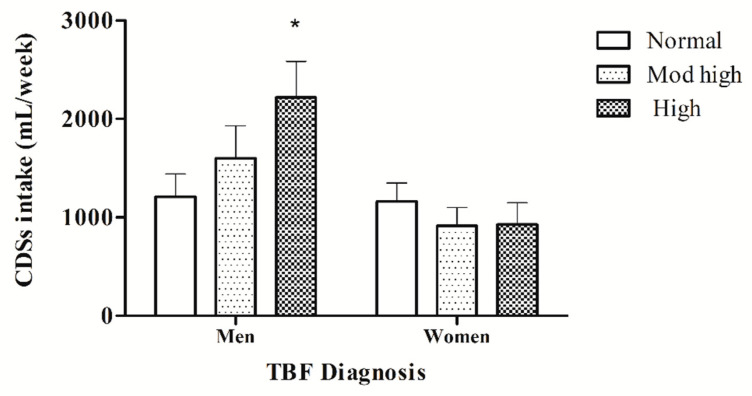
CSD consumption based on TBF% diagnosis. CSD intake is reported as the mean ± standard error of each group differentiated by a diagnosis of TBF% (normal, moderately high, and high) between men and woman. * *p* < 0.05 compared to the normal TBF% group, as per one-way ANOVA.

When comparing the amount/week consumption of CSDs between men and women with either a normal, moderately high, and high TBF%, a significant statistical difference was found between those men with a high TBF% compared with those with a normal TBF%. This was not observed in women (Figure 1). These results indicate that the higher amount/week of CSD consumption is found in those young men with the highest TBF%.

### 3.6. Comparing TBF% between the Quartiles of the Amount/Week CSD Consumption

**Figure 2:**
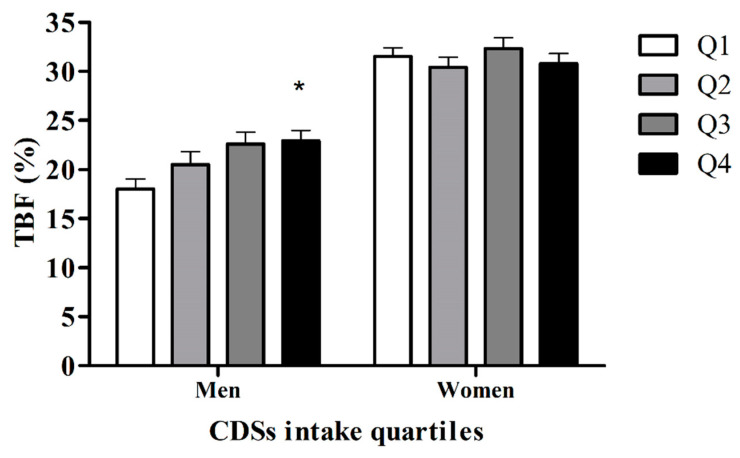
TBF% in men and woman subjects classified by the amount per week CSD consumption (Q1 is the lowest amount while Q4 is the highest amount). * *p* < 0.05 compared to Quartile 1, as per one-way ANOVA.

Using quartiles analysis to classify our subjects in relation to the amount/week of CSD consumption (Q1: ≤126 mL/week; Q2: 127–791 mL/week; Q3: 792–1456 mL/week; and Q4: ≥1457 mL/week), we found that only men were classified with the highest amount per week CSD consumption (Q4), and were those with a significantly higher TBF% (Figure 2).

### 3.7. Prediction Model

The coefficients obtained from the bivariate regression model was not statistically significant for most of the variables measured in the present study, except for TBF% and LDL in men, where it was found that CSD consumption accounts for 4% and 3% of the variance, respectively (Table 4).

## 4. Discussion

The majority of participants in the present study actively consume SDs and CSDs are clearly consumed more. Particularly, men consume significantly more than women. Interestingly, NCSDs are consumed at a significantly lower rate, possibly showing that awareness of the hazards of CSD consumption prompts individuals to choose the non-caloric version. The cola flavor is the one with the highest consumption in our population, consistent with a previous report [35]. Consumption of SSBs worldwide average 0.58 servings/day (an 8 oz serving), and the population with the highest intake were also men between 20 and 39 years [43]. In one such study, it was found that in Central Latin America, which includes Mexico, the average consumption was 0.8 servings/day. This is consistent with our present results and strengthens the validity of our SDsIQ. After converting our results to 8 oz per serving, the average CSD consumption for men was 0.9 servings/day and 0.6 servings/day in women, clearly consistent with previous findings. 

The caloric load attributable to the consumption of CSDs in participants would amount to approximately 600 kcal in men and 400 kcal in women weekly [7,8,9]. However, this amount of CSD consumption is significantly higher (~890 kcal) in men classified with a high TBF%. Importantly, the association of CSD consumption with a high TBF% in men was found through different analyses here: firstly, a significant positive correlation between TBF% with the amount/week of CSD consumption was found only in men, which also positively correlates with other anthropometric variables and glucose in men. Secondly, when classifying subjects by their TBF% diagnosis, it was clear that CSD consumption is significantly higher in men with a high TBF% diagnosis; also, when the classification was done with the quartiles of CSD consumption, those men with the highest consumption present a significantly higher TBF%, and finally the regression model revealed that CSD consumption was predictive of TBF%. An increase in TBF% per se has been associated with metabolic abnormalities [44,45], for which we can state that a high consumption of CSDs represents a metabolic risk factor, especially because the CSD consumption predicts the TBF% in those young men that participate in our study, and this result is more alarming if we consider that these measures are likely to increase when the subjects reach their 40s [46,47].

Why are these young men more likely to accumulate TBF compared to our young woman subjects? Sexual differences explain the anthropometric differences found here between men and women, as men present less TBF and greater amount of VF, while premenopausal women have greater subcutaneous femoral/gluteal fat [48,49]. These different fat distributions can be attributable in part to the different endocrine profiles between men and woman, where it is particularly clear that estrogens (ES) play a prominent role [50]. Man and woman produce both androgens and ES; androgens are higher in men and ES in women and it is well established that premenopausal women in general have a lower prevalence of metabolic disease [51], and VF has a higher association with metabolic disorders than subcutaneous fat due to the protective effect of gonadal hormones in a woman’s metabolism [52,53]. ES also regulates fat metabolism by promoting lipolysis and thermogenesis in brown adipose tissue [54]. Visceral adipose tissue express ES receptors (ERs) and these receptors trigger lipolysis via the activation of hormone-sensitive lipase, reducing VF accumulation [55]. This protective effect of ES is more evident in women than in men who, as found here, accumulates more VF; this accumulation of fat in our young men population can also be related with sexual differences in lipid and lipoprotein metabolism [56]. Here, we found a significantly higher amount of VLDL in men, while HDL was significantly higher in woman; these differences are consistent with previous findings, indicating that endocrine and lipoprotein metabolism signals interact to provide different metabolic backgrounds between men and woman. VLDL and HDL along with other signals act together in a complex system that orchestrates the genesis, accumulation, and catabolism of body fat. The different biological profiles and adaptations between men and woman make our young Mexican men population more vulnerable to metabolic abnormalities associated with high CSD consumption.

In woman, we found a mild but significant relation of TG and VLDL with CSD consumption; however, the increase in these metabolic measures has been related with atherosclerotic cardiovascular disease (ASCVD) in epidemiologic and genetic studies [57] and with stress in one experimental study [58], which is congruent with recent findings from our group (in preparation), indicating that woman, and not men, showed a significantly higher perceived stress associated with CSD consumption. This possible association suggests that men and woman may have different stress coping strategies and that the impact of CSD consumption in their metabolism is expressed differently. It is important to note that a low proportion of subjects (mean < 15%) showed that these metabolic measures where over the healthy range limits as classified by the WHO [36], which is congruent with the fact that the sample was a healthy and young population.

Sex differences in CSD intake and its effect in anthropometric measures found in the present study are of prominent relevance, suggesting that either sex or gender impact differently in the way this healthy population deal with the consumption of those commercially available products. Gender determines different roles in human society, and these roles and attitudes toward eating differ among the different cultures [59,60]. In the United States and Spain, women crave more sweet foods than men, the latter craving mainly savory foods [61]. Culture-associated habits may play an important role but remains to be explored, such as how much and which cultural differences actually account for the gender differences in CSD intake found here.

Biologically, men are generally larger and eat more than women, and meal size rather than frequency appear to underlie this difference [62]; also, eating stimulation by fasting is greater in men than women [63]. Women present a significantly higher TBF% than males, 26.0% vs. 13.0%, respectively, in normal “homeostatic eating-BMI of 22–23” adults [64]. In agreement, here woman present 31.3% TBF and men 21.13% (*p* < 0.01); note they are higher than Gallagher’s [64] older subjects, with similar anthropometrics. As mentioned before, men store more VF, whereas women store more fat in the gluteal–femoral region [56], and here men present 2.7% VF, while woman showed 2.0% VF (*p* < 0.01). Nevertheless, studies in a Western population from different ethnicities in the USA and New Zealand failed to detect gender differences in CSD intake [65,66,67,68], contrasting with the present results obtained in a young, “healthy” Mexican population, were sex differences in the amount of CSD intake were found to be highly significant. Note, however, that when grouped according to the TBF% diagnosis (normal, moderately high, and high), gender differences are found to be driven by the high TBF% subjects (see Figure 1, congruent with findings showing that sex differences in food preferences (high-fat and high-protein foods in men, and high-fat, high-sugar, and/or high carbohydrate foods in woman) are observed in the obese but not in the non-obese population [69,70].

Sex differences have been observed in the prevalence of obesity, high fat accumulation, and disordered eating [71]. In the brain, these may be explained by two different biological adaptations to the gonadal hormones: one is a relatively permanent “organizational effects”, also known as brain feminization or masculinization, occurring predominantly during puberty, and secondly by reversible “activational effects” occurring during the different biological cycles and upon the presence of stimuli inducing gonadal-hormone secretion [72]. Gustatory sensation and orosensory hedonics are shaped by these hormones and the hypothalamic–pituitary–gonadal (HPG) axis is one of their primary targets [73]; however, these hormones can also shape the brain’s reward circuits [74] related to hedonics and sensation, which can even overwrite the signals from the HPG axis that controls homeostatic eating behavior [75].

A vast amount of literature has revealed the biological mechanisms through which ES are anorexigenic [54,73]. However, little is known about the mechanisms through which androgens are orexigenic [54,76]. ES increase insulin secretion and sensitivity, and suppress gluconeogenesis metabolism [77,78]. Pro-opiomelanocortin (POMC) neurons in the arcuate (ARC) nucleus of the hypothalamus, providing anorexigenic activity, express ERs. In the nucleus of the solitary tract (NTS), which integrates visceral and satiety signals, ERs are highly expressed, and here ES controls the meal size, food intake, and body weight [62].

Sexual differentiation of the brain during puberty is considered an “organizational effect” of gonadal hormones, where testosterone has a transitory priming effect early after birth and during puberty, gradually rising again to promote brain masculinization [79]. Interestingly, it has been proposed that disordered eating is induced by organizational effects of gonadal steroid hormones [80], but it is important to note that food availability during sex differentiation shapes the juvenile transcriptome [81]. Moreover, androgen expression is regulated by dietary patterns [82] and these dietary patterns as well as food selection produce epigenetic changes that modify the expression of several transcripts [83,84], including gonadal [85]. For these reasons, it is important to study and understand better the epigenetic changes driven by food selection and the historic patterns of consumption, combined with the organizational effects that may also be modified by the individual dietary patterns and food and drinks selection [86].

Regarding gustatory sensation, women generally have more fungiform taste papillae than men [87], and the neural activity induced by taste stimulation stimulates more the parabrachial nucleus of the pons (PBN) in female compared to male rats, and the response magnitude (spike/s) after stimulation is significantly higher in females after sucrose and saccharine. Females had more “sweet-oriented” neurons than males [88]. In contrast, sweet and salt stimuli elicited more activity in the thalamic taste nucleus of male than female rats and the excitatory responses to taste stimuli is larger in female rat neurons [89]. This explains why men and woman perceived different saliencies of the stimuli, programed by both epigenetic associated adaptations and/or the organizational effects of the gonadal hormones, and both can be modulated by dietary patterns and food and drink selection.

Flavor hedonics depends on the brain reward system (BRS), composed of a complex set of neural networks that also regulate hunger, satiation, and/or food and drinks cravings, as well as many other pleasure experiences [90,91]. Orosensory hedonics and palatability are associated with it, and results from a brain evaluation of stimuli responses showed it produces the subjective experiences of pleasure, excess, and disgust to food and beverages. Increase flavor intensity enhances palatability coded by the insular cortex and amygdala BRS circuits [92,93,94], where nutrient, endocrine (POMC and ARC Hypothalamic nuclei), visceral-autonomic NTS, gustatory, and olfactory PNP information are integrated [95,96,97].

Craving intensity and the experience of pleasure are modulated by a complex interplay between dopamine (DOPA) and serotonin (5-HT) signaling in the BRS [98]. DOPA plays a role in cravings [99], while 5-HT regulates waiting [100], and both impact satiation and food reward [101]. Importantly ES modulates both the DOPA and 5-HT neural networks, influencing food intake [78,101]. The DOPA and 5-HT interplay regulated by ES may be important in optimizing the complex set of signals underlying the hedonic experience, controlling the amount and frequency of food and drinks intake [102].

In our sample, women drink less CSDs and do not show anthropometric or clear metabolic changes associated with its consumption, possibly due to the protective effect of ES [73,103]. However, this protection may not last for long, given that HFCS consumption can lead to ES expression derangements [104], so the apparent protection that may be attributable to ES in our sample may not prevail in woman adulthood. Another factor that can explain the lower consumption in women is an underestimation on the self-reported SDsIQ, or the sociocultural factors associated with women. The above should be evaluated in future research by complementing the applied questionnaires in this study with 24-h beverage consumption questionnaires and a food consumption diary to strengthen the validity of the amounts reported here. However, the consumption amounts are similar to those found in previous reports in a young Mexican population [105].

It is important to point out that this study was carried out with a sample of university students, which may have different characteristics from the general population of the same age range; for example, an academic degree can influence aspects such as physical activity and knowledge about processed foods. In addition, a university lifestyle can promote the development of smoking habits and stress, which influences the consumption of SSBs and foods in general; therefore, the results presented here may not be representative of the young population in general. The amount of sugar and artificial sweeteners from processed foods and other types of SSBs was not measured in our SDsIQ, and so the associations observed between CSD consumption and the anthropometric and metabolic variables should not be considered causal given the cross-sectional design used in the present study. SD consumption estimated from a self-reported questionnaire may present some inaccuracies; also, other circulating markers of metabolic importance, such as the free fatty acid or amino acid profiles, were not measured in this study. Finally, an analysis by subdivision of visceral tissue was not carried out.

## 5. Conclusions

In this apparently healthy, young, Mexican men population, we found a significant relation between the amount/week of CSD consumption and higher anthropometric measures, particularly TBF%. This higher amount of TBF represents a health risk, given that a progressive increase in this variable is likely to result in the development of metabolic abnormalities, leading to medical pathologies such as non-communicable chronic diseases [9,104], but may also be associated with the alteration of both the brain energy homeostasis system and the brain reward system, leading also to the development of toxic behavioral habits as well as psychiatric conditions such as depression and anxiety [97]. We predict that those men subjects with a high CSD consumption, associated with a high TBF%, can develop these types of diseases, which are likely to occur in years ahead if they keep their patterns of CSD consumption. 

This study shows us that the types of measures in the SDsIQ should be expanded upon to include the whole dietary patterns and food and drinks selection, along with metabolic, endocrine, and other biological measures. Moreover, behavioral and cognitive tests combined with other psychometric tools, such as the perceived stress questionnaire and stress resilience questionnaire, can also be applied in a dynamic manner and have a high likelihood to provide us with important data that, when analyzed in its full complexity, can help us understand more deeply the interaction between food and drinks selection, dietary patterns, and the development of cognitive, behavioral, and metabolic risk factors, from where we can predict and, for instance, help prevent the development of mental and chronic diseases by means of nutritional advice and intervention.

## Figures and Tables

**Table 1:** Difference in flavor preferences for caloric soft drinks (CSDs) and non-caloric soft drinks (NCSDs) according to sex.

	Men (*n* = 158)	Women (*n* = 238)	
SD Flavor	mL/Week ^a^	mL/Week ^a^	*p*-Value ^b^
Cola	755.4 ± 1149.0	535.2 ± 1234.4	0.077
Apple	171.0 ± 441.6	143.3 ± 524.3	0.587
Orange	205.4 ± 667.6	101.4 ± 299.5	0.038
Lemon-Lime	244.8 ± 619.2	127.5 ± 333.2	0.015
Grapefruit	68.5 ± 156.9	70.8 ± 192.5	0.903
Grape	52.5 ± 185.7	24.1 ± 69.8	0.033
**CSD Intake**	1489.9 ± 1909	999.5 ± 1672.6	0.007
**NCSD Intake**	53.2 ± 436.4	104.5 ± 631.1	0.257
Total SD Intake	1543.1 ± 1829	1104.1 ± 1847.8	0.021

^a^ The values are presented as the mean ± standard deviation. ^b^ One-way analysis of variance.

**Table 2:** The anthropometric and metabolic characteristics of the participants according to sex.

Variable	Men (*n* = 158) ^a^	Women (*n* = 238) ^a^	*p*-Value ^b^
Weight (kg)	70.55 ± 12.80	59.71 ± 12.31	0.000
Height (cm)	171.35 ± 6.32	158.89 ± 8.24	0.000
BMI (kg/m^2^)	23.96 ± 4.14	23.49 ± 4.17	0.309
Waist (cm)	84.43 ± 10.54	77.92 ± 10.33	0.000
Hip (cm)	97.52 ± 7.50	97.88 ± 9.24	0.689
WHR	0.80 ± 0.22	0.75 ± 0.18	0.009
WHTR	0.46 ± 0.13	0.46 ± 0.13	0.918
TBF (%)	21.35 ± 7.37	31.13 ± 7.57	0.000
VF (%)	2.75 ± 1.92	2.01 ± 1.82	0.000
Glucose (mg/dL)	84.98 ± 8.96	82.05 ± 6.64	0.000
TG (mg/dL)	97.10 ± 69.04	80.20 ± 42.72	0.004
TC (mg/dL)	165.91 ± 33.85	166.03 ± 29.40	0.971
HDL (mg/dL)	49.53 ± 15.28	53.62 ± 19.57	0.032
VLDL (mg/dL)	19.12 ± 13.88	15.90 ± 8.64	0.006
LDL (mg/dL)	94.74 ± 29.78	95.74 ± 30.13	0.752

**^a^** The values are presented as the mean ± standard deviation. **^b^** One-way analysis of variance.

**Table 3:** Correlations between the anthropometric and metabolic variables and CSD consumption according to sex.

Variable	Men (*n* = 158)	Women (*n* = 238)
Weight (kg)	0.130	−0.076
Height (cm)	−0.153	−0.084
BMI (kg/m^2^)	0.248 **	−0.051
Waist (cm)	0.243 **	−0.053
Hip (cm)	0.251 **	−0.074
WHR	0.177 *	0.030
WHTR	0.298 **	−0.048
TBF (%)	0.288 **	−0.023
VF (%)	0.261 **	0.029
Glucose (mg/dL)	0.189 *	0.108
TG (mg/dL)	0.028	0.147 *
TC (mg/dL)	0.072	−0.011
HDL (mg/dL)	−0.075	−0.041
VLDL (mg/dL)	0.047	0.154 *
LDL (mg/dL)	0.147	0.014

Two-tailed Spearman correlation. * *p* < 0.05 ** *p* < 0.01.

**Table 4:** Predictive models of caloric soft drink intake on the anthropometric and metabolic variables of metabolic risk in males and females, using bivariate regression analysis.

	Males (*n* = 158)		Females (*n* = 238)	
Variable	Β ± SE	*p*-Value	Β ± SE	*p*-Value
Weight (kg)	0.122 ± 0.001	0.59	−0.004 ± 0.001	0.953
BMI (kg/m^2^)	0.167 ± 0.000	0.053	0.000 ± 0.000	0.998
Waist (cm)	0.156 ± 0.000	0.061	0.002 ± 0.000	0.982
Hip (cm)	0.14 ± 0.000	0.091	−0.013 ± 0.000	0.847
TBF (%)	0.199 ± 0.000	0.021	0.018 ± 0.000	0.667
VF (%)	0.14 ± 0.000	0.106	0.029 ± 0.000	0.669
Glucose (mg/dL)	0.115 ± 0.000	0.166	0.074 ± 0.000	0.265
TG (mg/dL)	−0.014 ± 0.003	0.87	0.034 ± 0.002	0.611
TC (mg/dL)	0.065 ± 0.001	0.434	0.095 ± 0.001	0.155
HDL (mg/dL)	−0.081 ± 0.000	0.33	−0.041 ± 0.001	0.538
VLDL (mg/dL)	0.4 ± 0.001	0.963	0.04 ± 0.000	0.549
LDL (mg/dL)	0.169 ± 0.001	0.04	0.12 ± 0.001	0.07
